# Anti-Hepatitis B Virus Activity of Chickweed [*Stellaria media* (L.) Vill.] Extracts in HepG2.2.15 Cells

**DOI:** 10.3390/molecules17078633

**Published:** 2012-07-18

**Authors:** Lihua Ma, Jie Song, Yaqin Shi, Changmei Wang, Bin Chen, Donghao Xie, Xiaobin Jia

**Affiliations:** 1 Key Laboratory of New Drug Delivery System of Chinese Materia Medica, Jiangsu Provincial Academy of Chinese Medicine, 100 Shizi Street, Nanjing 210028, China; Email: huama322@163.com (L.M.); momo198420@hotmail.com (J.S.); shiyaqin1988@126.com (Y.S.); hanxi186@yahoo.com.cn (C.W.); jeromechen@126.com (B.C.); 2 School of Pharmacy, Jiangsu University, Zhenjiang, Jiangsu, 212013, China; Email: xiedonghao@126.com

**Keywords:** anti-hepatitis B virus activity, *Stellaria media* (L.) Vill., flavonoid, polysaccharide

## Abstract

*Stellaria media* (Linn.) Villars is a traditional Chinese medicine that has been used for over 200 years, mainly for the treatment of dermatitis and other skin diseases. It has also been used as an anti-viral agent. All the fresh chickweed juice samples used in this study were prepared using macroporous resin and ultrafiltration technology. The anti-hepatitis B virus (HBV) activity of *S. media* was evaluated *in vitro* using the human HBV-transfected liver cell line HepG2.2.15. The concentrations of hepatitis B surface antigen (HBsAg) and hepatitis B e antigen (HBeAg) in HepG2.2.15 cell culture medium were determined by enzyme-linked immunosorbent assay (ELISA) after *S. media*-n (SM-n) treatment for 6 or 9 days. HBV DNA was quantified using transcription-mediated amplification and real-time polymerase chain reaction. In HepG2.2.15 cells, 30 μg/mL SM-3 effectively suppressed the secretion of HBsAg and HBeAg with inhibition rates of 27.92% and 25.35% after 6 days of treatment, respectively. Consistent with the reduction in HBV antigens, SM-3 also reduced the level of HBV DNA in a dose-dependent manner. The characterization and quantitation of the chemical composition of SM-3 showed the presence of flavonoid C-glycosides, polysaccharides, and protein, which exhibited diverse antiviral activities. In conclusion, our results demonstrate that SM-3 possesses potential anti-HBV activity *in vitro*. This is the first report demonstrating the anti-HBV effects of *S. media*, which is currently under early development as a potential anti-HBV drug candidate.

## 1. Introduction

Hepatitis B virus (HBV) infection frequently results in both acute and chronic hepatitis and remains a major health problem worldwide. According to WHO estimates, over five million cases of acute hepatitis B infection occur annually and more than 350 million people suffer from chronic HBV infection [1]. Annually, HBV infection accounts for one million deaths worldwide, mainly due to cirrhosis, liver failure, and hepatocellular carcinoma [2]. Currently, lamivudine (3TC), entecavir [3], adefovir, telbivudine [4], IFN-α, and Peg-IFNα-2a [5] have been licensed globally for the treatment of HBV. Significant side effects of these drugs and inevitable drug resistance have been noted. The use of nucleoside analogues for the treatment of HBV also has disadvantages, such as the requirement for long-term therapy and high-drug resistance rate [6,7]. Furthermore, these agents are expensive [8,9]. A significant unmet medical need exists for new safe and efficacious anti-HBV drugs [10]. However, to develop new anti-HBV agents remains a significant challenge. Given the well-known potency of Chinese herbs in the treatment of diverse diseases, we were interested in studying their potential anti-HBV activity.

*Stellaria media* (L.) Vill., commonly known as chickweed, is a Chinese folk medicine that belongs to the Caryophyllaceae flowering plant family, which characteristically contain typical C-glycosyl-flavones [1]. This plant is distributed widely throughout China and contains many polysaccharides, flavonoids, cyclic peptides as well as other compounds, which exhibit extremely effective anti-inflammatory and antiviral activity [11]. It has also been used as a folk medicine; for example, the 17th century herbalist John Gerard recommended it as a remedy for mange [12]. Modern herbalists prescribe it mainly for skin diseases, but also for bronchitis, rheumatic pains, arthritis, and period pain [13]. A poultice of chickweed can be applied to cuts, burns, and bruises [14]. However, not all these uses are supported by scientific evidence. In this study, our results show that chickweed that has undergone membrane ultrafiltration possesses excellent anti-HBV activity. We also report the presence of large quantities of polyphenol and macromolecular compounds that may contribute to the HBV inhibitory effect of chickweed. This is the first report describing the anti-HBV activity of *S. media*, with great significance in research investigating the underlying mechanism of this activity.

## 2. Results and Discussion

### 2.1. Cytotoxic Effects of Different Stellaria Extracts

*S. media* was collected from the Nanjing Botanical Garden, Memorial, Sun Yat-Sen (China) during its flowering period. The plant is native to Europe, and is both edible and nutritious; it is used as a raw leaf vegetable in salads. Prior to this study, no report has investigated whether *S. media* can inhibit HBV. The HepG2.2.15 cell line was established using a gene plasmid containing two heads and tails attached to the adw subtype of HBV transfected into human hepatoma cell lines. This cell line has a consistently high level of expression of HBsAg and HBeAg, and exhibits the biological activity of the virus particles *in vitro* [15].

**Figure 1:**
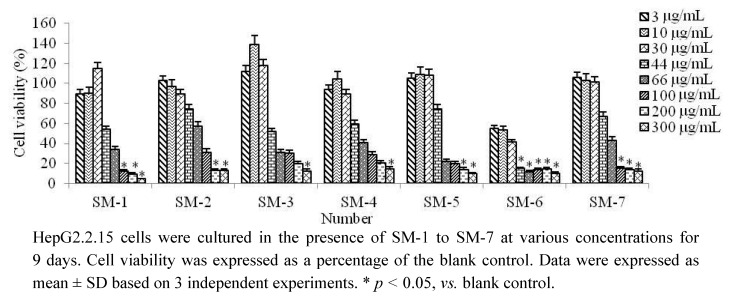
MTT cytotoxicity assay results for the seven Stellaria media samples.

The viability of the HepG2.2.15 cells in the presence of various concentrations of different *S. media* extracts was examined using an MTT assay. The results showed that all sample concentrations below 30 μg/mL had no significant toxicity in HepG2.2.15 cells at 9 days (Figure 1). The cytotoxicity of these samples was examined to determine the treatment concentrations in the following HepG2.2.15 cell culture experiments.

### 2.2. Anti-HBV Antigens Secretion Activity in HepG2.2.15 Cells after Stellaria Treatment

The HepG2.2.15 cells were treated with SM-n at various concentrations to determine their inhibitory effects on the secretion of HBsAg and HBeAg after 9 days. The antigens in the culture supernatants were quantified using specific ELISA kits. The anti-HBV activity of each sample was shown by its inhibition of antigen secretion in HepG2.2.15 cells after treatment with the corresponding sample. 3TC was used for positive cytotoxicity control. The data demonstrated that SM-3 significantly reduced HBsAg and HBeAg secretion, albeit to a slightly lesser degree (Table 1). Notably, for both HBsAg and HBeAg, no significant difference was observed between SM-3 and 3TC.

In HepG2.2.15 cells, SM-3 showed no inhibitory effect on cell proliferation at concentrations of up to 30 μg/mL, as shown by the MTT assay. The 50% cytotoxic concentration was determined to be 38 μg/mL. These results were used to determine the dose range of SM-3 for subsequent experiments.

**Figure 2:**
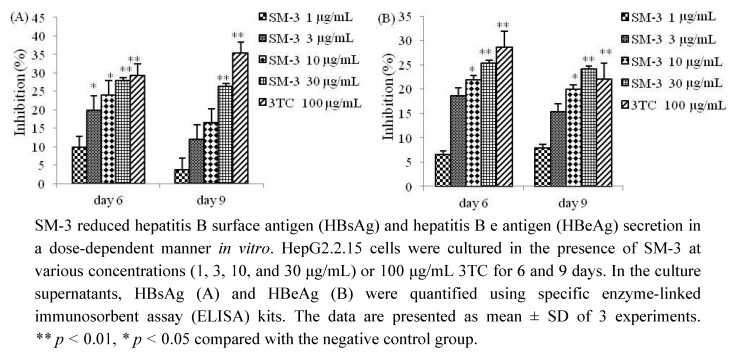
Inhibition of HBsAg and HBeAg secretion by SM-3 and 3TC.

SM-3 effectively suppressed the secretion of HBV antigens from HBV-transfected HepG2.2.15 cells, achieving 9.83%, 19.79%, 24.05%, and 27.92% inhibition of the secretion of HBsAg, and 6.51%, 18.55%, 21.83%, and 25.35% inhibition for HBeAg, at 1, 3, 10, and 30 μg/mL after 6 days of treatment (Figure 2). In the same experiment, 100 μg/mL 3TC also suppressed the secretion of both HBsAg and HBeAg, achieving 29.31% and 28.63% inhibition, respectively (Figure 2). However, the secretion of HBV antigen at 9 days was not increased compared with those at 6 days, and this study only assayed the secretion of HBV antigens after 6 and 9 days (Figure 2). These data suggest that the inhibitory activity of SM-3 on the secretion of HBV antigens is similar to that of 3TC.

**Table 1:** Comparison of the anti-HBV activity demonstrated by different *S. media* components.

Sample	Concentration (μg/mL)	HBsAg (%)	HBeAg (%)
SM-1	30	27.35	11.37
SM-2	30	11.42	5.78
**SM-3**	**30**	**34.15**	**27.28**
SM-4	30	13.69	4.59
SM-5	10	25.76	9.18
SM-6	10	10.43	5.12
SM-7	30	23.64	12.37
**3TC**	**100**	**38.65**	**33.56**

HBsAg, hepatitis B surface antigen; HBeAg, hepatitis B e antigen.

### 2.3. Effects of SM-3 on HBV DNA Expression

**Figure 3:**
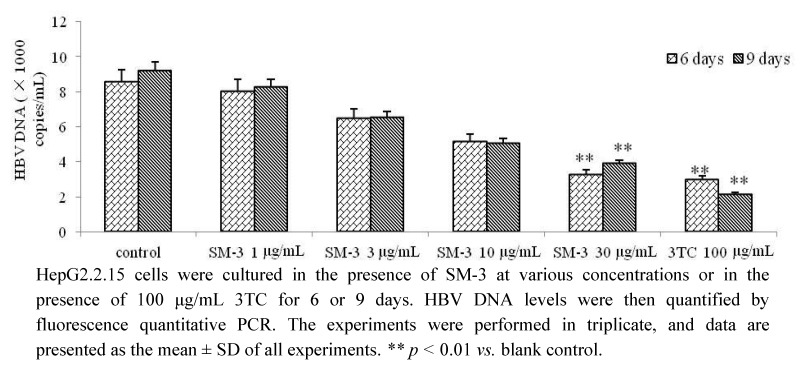
Inhibitory effect of SM-3 on HBV DNA levels in HepG2.2.15 cells.

HBV is a DNA virus and HBV DNA testing is the most accurate indicator for measuring the contagiousness of hepatitis B. HBV DNA load is an important indicator of the efficacy of the antiviral treatment selected, and its accurate detection during antiviral therapy is important for guiding clinical decisions [16]. The cytotoxicity of the samples was examined to determine the treatment concentrations to be used in the HepG2.2.15 cell culture experiments. According to the analysis of the pre-test results, only SM-3 was able to significantly reduce HBsAg and HBeAg secretion. To further confirm the anti-HBV activity of SM-3 in HepG2.2.15 cells, the effect of SM-3 treatment on the level of HBV DNA was evaluated. Consistent with the inhibitory effects on HBsAg and HBeAg secretion, 30 μg/mL SM-3 treatment led to a statistically significant reduction in the level of extracellular HBV DNA compared with the negative control after 6 and 9 days (Figure 3).

### 2.4. Chemical Characterization of SM-3 and Analysis of Total Sugar, Protein, and Total Flavonoid Content

**Figure 4:**
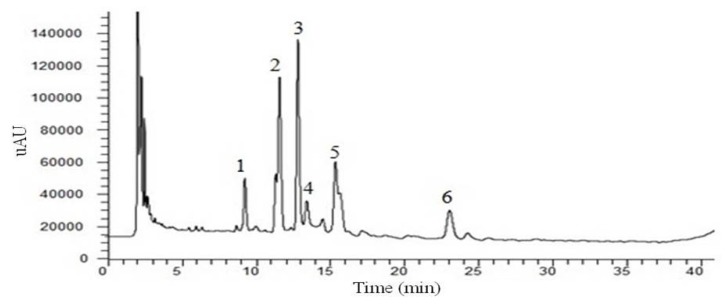
Chromatogram of SM-3 after elution with ethanol at 330 nm.

Under the guidance of the “component structural theory”, we performed a qualitative study on the chemical composition of SM-3. Total sugar, protein, and total flavonoid content were determined by a UV spectrophotometer, with the ratio of their content found to be total flavonoid:total sugar:protein = 1:11.86:16.70. This analysis was performed to reveal the chemical composition of SM-3 at the component level. The primary structure and antiviral activity of the macromolecular compounds, mainly referring to the polysaccharides were analysed in our laboratory (manuscript in preparation). The analysis results of SM-3 by HPLC demonstrated that the compounds with UV absorption were mainly flavonoids (Figure 4). 

**Figure 5:**
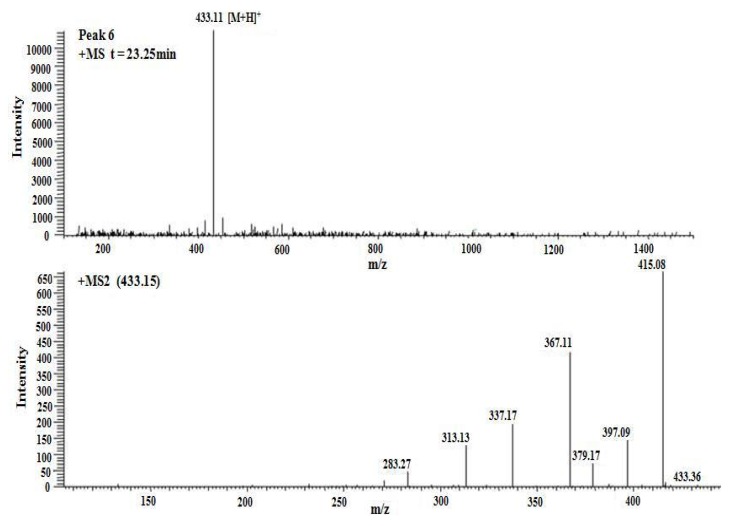
Positive ion ESI mass spectra and MS^n^ spectra of peak 6.

**Figure 6:**
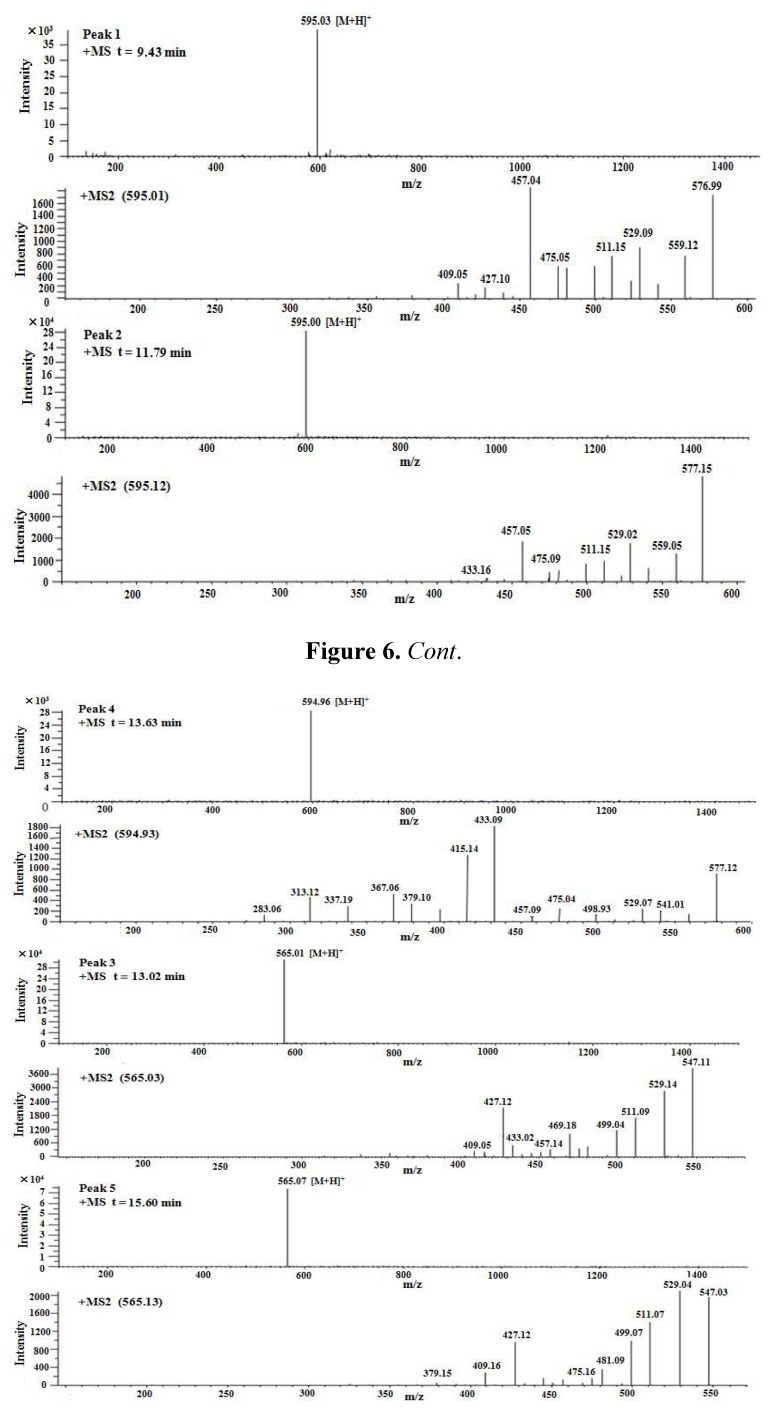
Positive ion ESI mass spectra and MS^n^ spectra of peak 1, 2, 4, 3, 5.

To further analyze the flavonoid qualitatively, SM-3 was analysed by LC/MS in positive ion mode. Data obtained during the LC-MS3 experiments demonstrated that the fragmentation regularity of peak 6 was consistent with vitexin [M+H]^+^ 433.11 (Figure 5), which has been reported previously [17]. Apart from this, other peaks did not permit the structures to be identified easily; however, the structures of peaks 1, 2, and 4 were flavone C-glycosides and apigenin connected to two 6-carbon sugars ([M+H]^+^ 595, Figure 6), and the structures of peaks 3 and 5 were flavone C-glycosides and apigenin connected to one 6-carbon sugar and one 5-carbon sugar ([M+H]^+^ 565, Figure 6).

**Figure 7:**
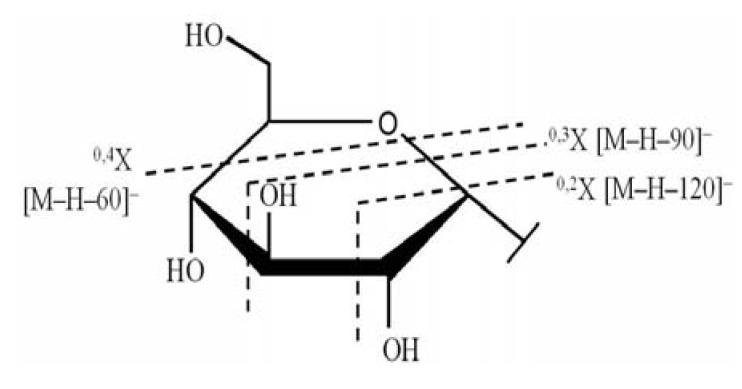
The fragmentation regularity of flavone C-glycosides sugar ring.

Our results on the regularity of the fragmentation process confirm the findings from previous analysis [18] and data were consistent with the fragmentation regularity of flavone C-glycosides (Figure 7). These compounds were identified as isomers according to the above results. However, their chemical structure could not currently be determined by LC/MS. Therefore, the flavonoids in SM-3 should be further separated, purified, and analysed.

### 2.5. Anti-HBV Efficacy Validation of the Components of SM-3

**Figure 8:**
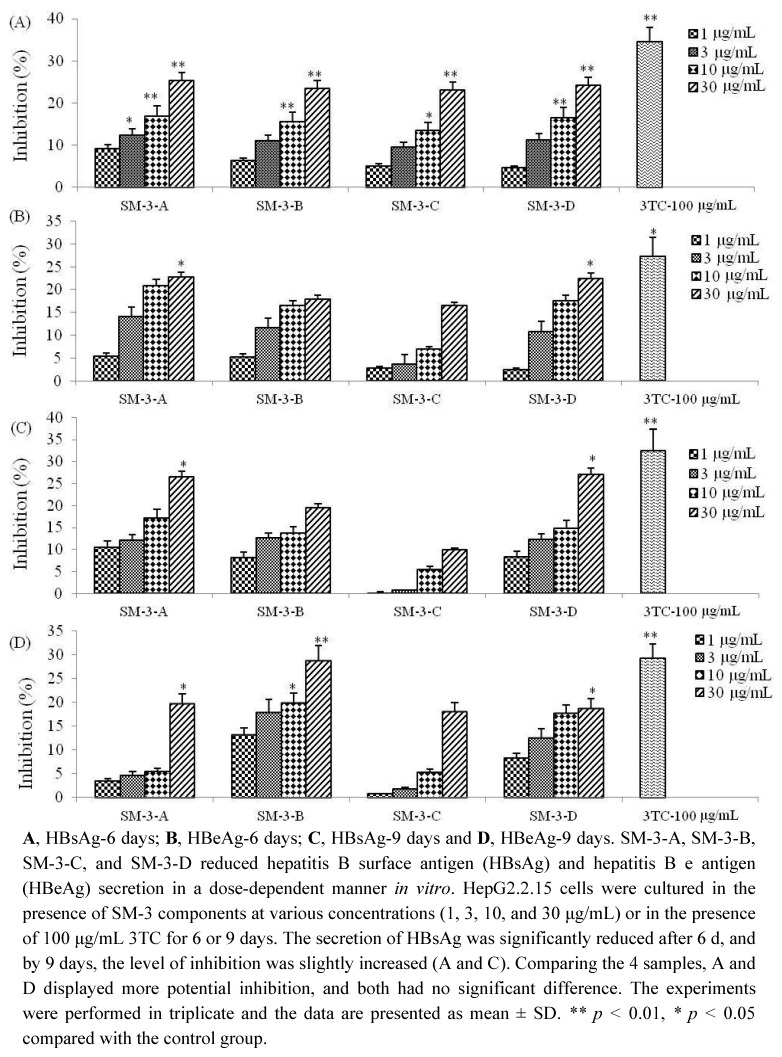
Inhibition of HBsAg and HBeAg secretion by SM-3 and 3TC.

To further verify the anti-HBV activity of SM-3, the sample was treated with macroporous adsorption resin to divide it into its aqueous and alcohol soluble parts. The chemical analysis of SM-3 showed that it contained two types of compounds: polyphenolic and macromolecular substances. Samples were obtained by mixing the same amount of two parts of the crude extract. These samples were named as SM-3-A, SM-3-B, SM-3-C, and SM-3-D. As shown in Figure 8, at 6 days, the inhibition ratios of the secretion of HBsAg and HBeAg by SM-3-A were 25.29% and 22.63%, respectively, compared with 24.14% and 22.42% by SM-3-D.

Statistically, the inhibition rates differences were not significantly different between the two samples, but they were lower than the positive control drug 3TC, with the inhibition of HBsAg and HBeAg being 34.49% and 27.29%, respectively. After 9 days, the inhibition rate of HBsAg was slightly increased; however, the inhibition rate of HBeAg showed the opposite trend. Interestingly, the anti-HBV effect of SM-3-B was very obvious, with a HBeAg inhibitory rate of 28.74%, which was worthy of further consideration. The data demonstrated that SM-3-A exhibited the best inhibitory effect of the four mixtures (Figure 8), which provided further validation of the “component structure theory”. Based on these results, the inhibitory activity of SM-3 appears to warrant further study.

Thus far, no report has been published on the anti-HBV activity of *S. media*. This study has shown that SM-3 has significant anti-HBV activity by detecting both antigens and HBV DNA. Large amounts of polyphenol compounds, such as flavonoids, were found in the SM-3 fraction. It is well known that polyphenols bind to proteins to form unstable complexes [19]. Therefore, enveloped viruses, such as HBV, may be affected by polyphenols given that this class of naturally occurring substances which might interact with the glycoproteins of the viral envelope. Moreover, other macromolecular compounds have been reported to exhibit an anti-HBV effect, including polysaccharides, which displayed anti-HBV activity by enhancing immune regulation and acting as antioxidants [20,21]. Together, these findings help to establish a theoretical foundation for the inhibitory effect of SM-3, and the material basis of its anti-HBV activity may result from an interaction of multiple components being present in a certain proportion.

## 3. Experimental

### 3.1. Compounds and Reagents

Fresh chickweed was collected from-Nanjing Botanical Garden Memorial Sun Yat-Sen, China, and repeatedly washed in water, with the final wash using distilled water. Species identification was conducted by Professor D.K. Wu from Nanjing University of Chinese Medicine, Nanjing, China. The vouchers of the authenticated samples were deposited at the Key Laboratory of New Drug Delivery System of Chinese Materia Medica, Jiangsu Provincial Academy of Chinese Medicine. 

**Figure 9:**
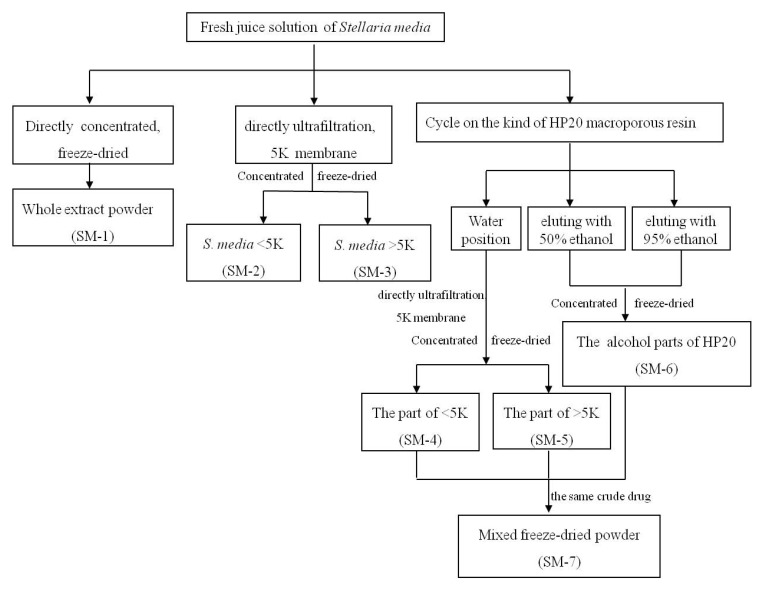
Flow chart of the preparation of the seven samples by different methods.

A total of 5 kg of chickweed was homogenised in 5,000 mL of distilled water at 25 °C using a domestic blender. The total homogenate was filtered through a NITEX nylon mesh filter and the filtrate was centrifuged at 5,000 ×g for 15 min at 25 °C. The samples were named as SM-1 to SM-7 after different preparation methods (Figure 9). The final filtrate was used directly after being centrifuged or lyophilised, and was reconstituted as needed into stock solutions of 100 mg/mL in water.

### 3.2. Cell Culture

Confluent cultures of HepG2.2.15 cells (Shanghai Bioleaf Biotech Ceo., Ltd., Shanghai, China) were treated with various doses of antiviral compounds in MEM (Sigma Chemical Co., St. Louis, MO, USA) supplemented with 10% fetal bovine serum (Gibco-Invitrogen, Carlsbad, CA, USA) and 500 μg/mL G418 (Sigma Chemical Co.) under 5% CO_2_ atmosphere at 37 °C. Fresh medium with the same concentration of compounds was replaced on day 4. Subconfluent monolayer cells of HepG2.2.15 were detached from the culture dishes by trypsin treatment, centrifuged for 5 min, and resuspended in fresh media. Cells were seeded in 96-well flat-bottom plates at a density of 5.0 × 10^4^ cells/well and grown in fresh medium. All samples used in this study were dissolved in PBS, and lamivudine (3TC) was used as the positive control. After plating for 24 h, the confluent HepG2.2.15 cells were fed with the medium containing the indicated concentration of test samples and fresh medium with the respective reagents was changed every 3 day. Cell viability was then analysed, or the culture media and cells were harvested for detection. Cell viability was calculated as follows:



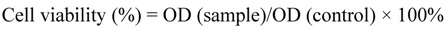



### 3.3. Cytotoxicity Assay

The 3-(4,5-dimethylthiazol-2-yl)-2,5-diphenyltetrazolium bromide (MTT, Sigma Chemical Co.) assay was used to measure the viability of the cultured cells and to further identify the non-toxic concentrations of different reagents to the culture cells [22]. The HepG2.2.15 cells were treated with different concentrations (300, 200, 100, 66, 44, 30, 10, and 3 μg/mL) of SM-n (n = 1, 2, 3,……8) for 72 or 144 h. 3TC (100 μg/mL) was used as the positive cytotoxicity control. Incubated medium was then removed and 100 μL of fresh medium containing MTT (2.5 mg dissolved in 50 μL of dimethyl sulphoxide [DMSO]) was added to each well. After incubation for 4 h at 37 °C, the culture medium containing MTT was removed, 100 μL DMSO was added to each well, and the viable cells were detected by measuring absorbance at 490 nm. Each experiment was performed in triplicate. Cell viability was expressed as a percentage of the control. Concentrations were considered non-toxic if the corresponding cell viability was >95%.

### 3.4. Measurement of HBV Antigens

HepG2.2.15 cells were seeded in 96-well plates at a density of 5.0 × 10^4^/well for the measurement of HBV antigens, and HBV DNA. After incubation with various concentrations of the samples, or 3TC for 6 or 9 d, the culture medium was collected, cell debris was removed, and the resulting sample was stored at −70 °C until further analysis. The levels of HBV surface antigen (HBsAg) and e antigen (HBeAg) in the supernatant of the HepG2.2.15 cells were determined using the enzyme-linked immunosorbent assay (ELISA) according to the manufacturer’s protocol (Nanjing Yingke Xinchuang Biotech Co., Ltd., Nanjing, China). Absorbance was measured at 450/630 nm using a microplate reader (Varioskan, Thermo Scientific, Vantaa, Finland).

### 3.5. Quantification of HBV DNA by Quantitative PCR

HepG2.2.15 cells were treated with antiviral agents as described above. For the intracellular assay, the cells were quantified with a HBV DNA quantitative kit. The primers specific for the detection of HBV DNA was: P1: 5′-ATCCTGCTGCTATGCCTCATCTT-3′, P2: 5′-CAGTGGGGAAAGCCCTA CAA-3′, and the sequence of the probe was 5′-TGGCTAGTTTACT AGTGCCATTTTG-3′. The kit was based on transcription- mediated amplification and a hybridization protection assay [23]. To isolate HBV DNA from the HepG2.2.15 cells, DNA was extracted from culture supernatants, according to the instructions of the DNA extraction kit used (CAS Array, Shanghai, China). In brief, a mixture of the cell culture supernatants or amplification standards and 50 μL of the reaction mixture containing 1.0 μmol/L primer, 0.1 μmol/L fluorescent probe (F Probe), 200 μmol/L DNA, 50 nkat Taq DNA polymerase, and 1× buffer were placed in a 0.2 mL reaction tube. The reaction tube was heated to 93 °C for 2 min pre-denaturation, then 93 °C for 45 s and 55 °C for 60 s, during the first 10 cycles, followed by 30 cycles at 93 °C for 30 s and 55 °C for 45 s. The copy number calibrators of 1 × 10^5^, 1 × 10^6^, 1 × 10^7^, and 1 × 10^8^/L were also amplified. The difference in fluorescence before and after amplification was plotted on the vertical axis, the copy number was plotted as the abscissa. The ABI7000 computer software automatically computed the standard curve and the initial copy number of samples, providing quantitative HBV DNA results. The HBV DNA test results were expressed using qualitative methods (negative: copy number of <10^6^ copies/L; positive: ≥10^6^ copies/L) [24].

### 3.6. Preliminary Analysis of the Composition of SM-3

The contents of the components in SM-3 were determined by using a UV-spectrophotometer, including total flavonoids, polysaccharides, and protein. The total content of the polysaccharides was determined by the phenol-sulphuric acid colorimetric method using glucose as the standard [25]. In addition, protein was quantified according to the Bradford method [26] using bovine serum albumin as the standard. Lastly, the total content of flavonoids was determined by UV using apigenin as the standard, and the characterization of their chemical composition was achieved by high-performance liquid chromatography (HPLC; Agilent 1100, Santa Clara, CA, USA) and liquid chromatography coupled to mass spectrometry with ion trap mass analyser (LC/MS; Thermo LXQ, ESI, Waltham, MA, USA). 

An Altima C_18_ (250 mm × 4.6 mm, 5 μm) column maintained at 35 °C was used. The solvents used were (A) 0.1% formic acid in water and (B) methanol. The elution gradient established was as follows: for 0–3 min, 75%–70% (A); 3–30 min, 70%–55% (A); 30–35 min, 55%–50% (A); and 35–40 min, 50%–75% (A), using a flow rate of 1 mL/min. All online detection was carried out in the diode-array detector (DAD), using 280 and 330 nm as the preferred wavelengths, and in a mass spectrometer (MS) connected to the HPLC system via the DAD cell outlet. MS detection was performed using a Thermo LXQ equipped with an electrospray ionization source and an ion trap mass analyser, which were controlled by the Chemstation software. The drying gas used was nitrogen at a flow rate of 80 mL/min at 350 °C. The source voltage was −3,500 V, the capillary voltage was −136 V, and skimmer voltage was −40 V. Spectra were recorded in negative ion mode between 100 and 1,500 *m/z*. The MS detector was programmed to perform a series of positive ion scans: A full scan and an MS-MS scan of the most abundant ion in the first scan, using a normalised collision energy of 1 V.

### 3.7. Statistical Analyses

All experiments were repeated at least three times, and the results were expressed as mean ± SD. Statistical significance was determined using the analysis of variance or a rank-sum test. Differences were considered to be statistically significant at *p <* 0.05.

## 4. Conclusions

In conclusion, this study demonstrates that SM-3 possesses potent anti-HBV activity *in vitro*. Fraction SM-3 was found to be more effective than other components of *S. media* at inhibiting the secretion of HBV antigens. The present findings suggest that *S. media* may be useful in the development of novel anti-HBV drugs.
